# Design and analysis of a field modulated magnetic screw for artificial heart

**DOI:** 10.1063/1.4975699

**Published:** 2017-02-06

**Authors:** Zhijian Ling, Jinghua Ji, Fangqun Wang, Fangfang Bian

**Affiliations:** School of Electrical and Information Engineering, Jiangsu University, Zhenjiang 212013, China and Jiangsu Key Laboratory of Drive and Intelligent Control for Electric Vehicle, Zhenjiang 212013, China

## Abstract

This paper proposes a new electromechanical energy conversion system, called Field Modulated Magnetic Screw (FMMS) as a high force density linear actuator for artificial heart. This device is based on the concept of magnetic screw and linear magnetic gear. The proposed FMMS consists of three parts with the outer and inner carrying the radially magnetized helically permanent-magnet (PM), and the intermediate having a set of helically ferromagnetic pole pieces, which modulate the magnetic fields produced by the PMs. The configuration of the newly designed FMMS is presented and its electromagnetic performances are analyzed by using the finite-element analysis, verifying the advantages of the proposed structure.

## I. INTRODUCTION

Nowadays, cardiovascular diseases have been a major cause of death. Transplantation of donor and implantation of artificial heart are the major treatment of terminal heart diseases. Due to the shortage of natural hearts, artificial heart (AH) is the best treatment option for many heart failure patients.^1^ The AH drive device must meet a variety of requirements, such as small size, high reliability, high power density and light weight.^2^ Existing electric drive based AHs employ rotary blood pumps but suffer from damage to blood cells.^3^ Linear AH motors were proposed to solve this problem, but its force density was relatively poor.^4^ Although double stator structure and vernier topology can improve the force capability of the linear AH motor, they suffer from difficult manufacture and low power density, respectively.^5,6^

Recently, a new magnetic screw with improved force density was proposed, which can convert rotation motion into linear motion.^7,8^ Compared with the mechanical lead screw, the magnetic screw has the merits of inherent overload protection, minimal friction, and low maintenance because of contact-free force transmission through the magnetic fields. The thrust force density of Field Modulated Magnetic Screw (FMMS) is much higher than the existing linear AH motor. The thrust force density per volume of the FMMS is approximately 20MN/m^3^, and the thrust force density of the existing linear motor is 300kN/m^3^.^9^ The advantage of the thrust force density is obviously. The major objective of this paper is to evaluate and confirm the advantage of the FMMS.

## II. STRUCTURE OF THE FMMS

**FIG. 1.**
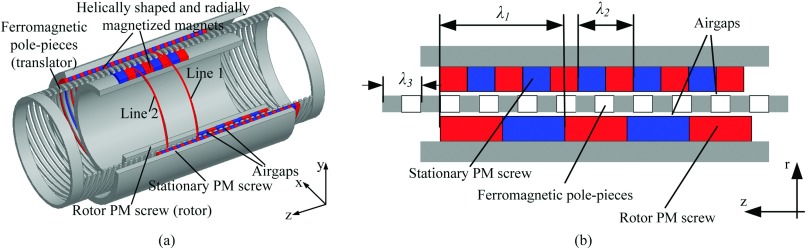
Configurations of FMMS. (a) 3D model. (b) 2D axially symmetric model.

Fig. 1(a) shows the structure of the proposed FMMS. It consists of three main parts: the outer and inner carrying the radially magnetized helically PMs and the intermediate having a set of helically ferromagnetic pole pieces, which modulate the magnetic fields produced by the PMs. The proposed FMMS offers the advantages of a high force density and large stroke. Due to the translator adopted the material of ferromagnetic pole-pieces, the FMMS reduced the consumption of the PMs obviously. The translator move back and forth along the Z-axis, and rotor rotate about the same axis. Since the helical PMs are placed on both the rotor screw and the stationary screw, the magnetic field distribution in the air-gap of the FMMS will be 3D. In order to simplify the thrust force calculation and predict the magnetic field distribution of the FMMS, the 2D axi-symmetric models of the FMMS are employed as shown in Fig. 1(b). The number of ferromagnetic pole-pieces is *n*_*s*_, *p*_*r*_ and *p*_*s*_ are the number of pole-pairs on the rotor screw and stationary screw, respectively.

$$n_{s}=p_{r}+p_{s}$$ (1)

As an example, a rotor magnetic screw with 4 pole-pairs and a linear distance defined by the lead $\lambda_{1}$ is 30mm, the translator with 13 pole-pairs and the $\lambda_{3}$ is 9mm, the stationary PM screw with 9 pole-pairs and the $\lambda_{2}$ is 13mm. The FMMS can convert the rotary speed *Ω* of 1625 r/min to the linear speed *V* of 1 m/s. Due to the introduced the concept of magnetic screw, the trans-rotary problem associated with the linear magnetic gear can be solved. The relationship between the translator force and the rotor torque can be simply obtained by assuming an ideal FMMS in which the power associated with the translator motion, *P*_*t*_, equals the power associated with the rotor rotation, *P*_*r*_, as shown in

$$P_{t}=P_{r}=F_{t}^{*}V=T_{r}^{*}\Omega$$ (2)

Therefore, the gear ratio *G* is obtained:

$$\frac{F_{t}}{T_{r}}=\frac{\Omega }{V}=G$$ (3)

## III. ANALYSIS AND EVALUATION

**FIG. 2.**
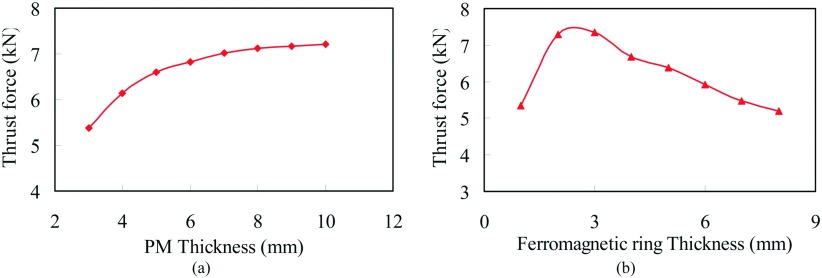
Variation of peak thrust force. (a) PM thickness. (b) Ferromagnetic ring thickness.

In order to demonstrate the proposed FMMS, the finite-element (FE) analysis has been employed for the design and analysis of the FMMS, This work would like to thank ANSYS for providing the FEA software.^10,11^ For the proposed FMMS, the thickness of PM and ferromagnetic pole-piece will significantly affect the electromagnetic thrust force. By increasing the thickness of the PM, the power of the PM is increased. Therefore, it is important to optimize the parameters. Fig. 2(a) shows the influence of PM thickness on thrust force. The increase of the PM thickness can enhance the thrust force. These indicate that, by increasing the PM thickness, the thrust force will rise until a certain point, after which it stagnates due to the magnetic density in the air-gap is saturation. Hence, based on the considerations of size and cost, the PM thickness is selected to be 6mm. The PMs are placed on the rotor screw and stationary screw must be equal. Intuitively speaking, confrontation of two sets of magnets with unequal thickness could result in demagnetization of the thinner magnets. Fig. 2(b) shows the variation of the thickness of ferromagnetic pole pieces. It is seen that as the thickness increases, the peak value of the thrust force reaches a maximum when the thickness of ferromagnetic pole pieces is 3mm.

**FIG. 3.**
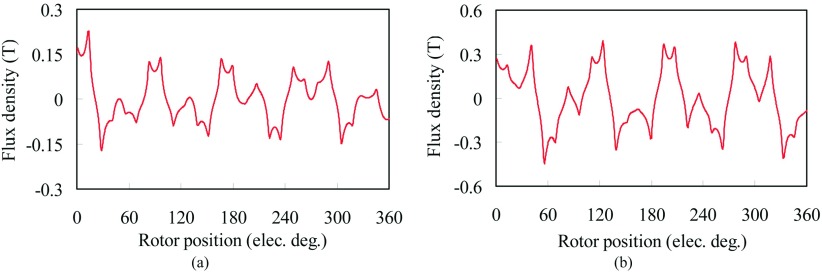
Radial flux density in the air-gap adjacent to the rotor screw due to stationary screw magnets. (a) Adjacent to the rotor screw. (b) Adjacent to the stationary screw.

In order to demonstrate the proposed FMMS, the electromagnetic characteristic of FMMS is calculated by finite-element analysis. The magnetic field distribution in the air-gap of the FMMS will be 3D, and in order to extract the radial flux density, the circle line 1 and circle line 2 have been proposed, as shown in Fig. 1(a). Fig. 3 shows the radial flux density in the air-gaps adjacent to the rotor screw and stationary screw, respectively. It can be seen that the introduction of the helical ferromagnetic pole pieces results in an asynchronous harmonic having the same number of poles as the PMs on the other armature.

**FIG. 4.**
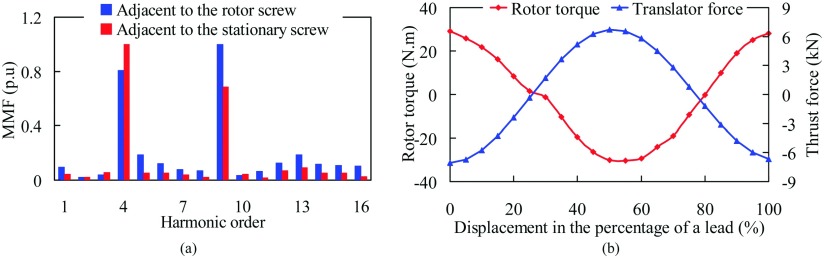
The operation principle of the FMMS. (a) The MMF space harmonics of the FMMS. (b) Variation of the rotor torque and translator thrust force.

To further investigate the radial flux density in the air-gaps, its MMF space harmonics is calculated, as shown in Fig. 4(a). There is a good agreement between the force-producing and torque-producing on each space harmonic spectra, suggesting that the assumption of the FMMS is acceptable. Also, the result of the torque and thrust force are also predicted, as compared in Fig. 4(b). It can be observed that the thrust force of translator is up to 6.1 kN and the torque of the rotor screw is 33.8 N.m. When the translator travels at 1 m/s, the rotate speed of the rotor screw is 1625 r/min. However, the power of the rotor rotation, *P*_*r*_ is 5% lower, which is due to the helical effect. The results also validate the thrust force *F*_*t*_ and torque *T*_*n*_ transmission ratio in (3), which satisfies the modulation ratio.

**FIG. 5.**
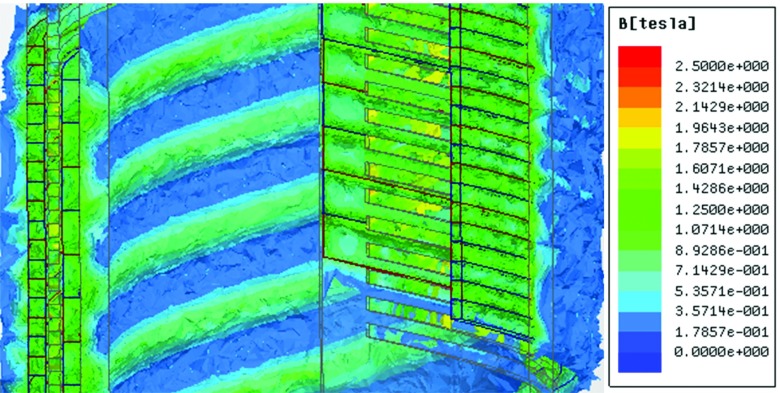
The flux density distribution of the FMMS.

Fig. 5 shows the magnitude of flux density distribution of the FMMS. By examining the flux density distribution, it can be observed that there is an ideal helical magnetic thread in the proposed FMMS. It should be noted that the flux density is not saturation, the level of the flux density is less than 2T. In the future study, the high-performance PM will used in the FMMS.

## IV. CONCLUSION

In this paper, a high force density linear actuator, based on the concept of magnetic screw and linear magnetic gear, has been described for AHs. The novelty of the proposed FMMS is introduced the ferromagnetic iron ring. The electromagnetic characteristic of FMMS is calculated by finite-element analysis. It can be seen from the finite-element results, the magnetic field modulated effect of the FMMS is acceptable. The proposed FMMS offers the advantages of a high force density and large stroke. Further research is required to explore with this methodology and design optimization of the FMMS.
